# The First Isolation and Molecular Characterization of Shiga Toxin-Producing Virulent Multi-Drug Resistant Atypical Enteropathogenic *Escherichia coli* O177 Serogroup From South African Cattle

**DOI:** 10.3389/fcimb.2019.00333

**Published:** 2019-09-24

**Authors:** Peter Kotsoana Montso, Victor Mlambo, Collins Njie Ateba

**Affiliations:** ^1^Bacteriophage Therapy and Phage Bio-control Laboratory, Department of Microbiology, Faculty of Natural and Agricultural Sciences, North-West University, Mmabatho, South Africa; ^2^Faculty of Agriculture and Natural Sciences School of Agricultural Sciences, University of Mpumalanga, Nelspruit, South Africa

**Keywords:** atypical enteropathogenic *E. coli* (aEPEC), bundle-forming pili (BFP), *E. coli* O177, virulence factors, antimicrobial resistance, shiga-toxins, diarrhoeagenic *E. coli*

## Abstract

Atypical enteropathogenic *E. coli* (aEPEC) is a group of diarrhoeagenic *Escherichia coli* with high diversity of serogroups, which lack the bundle-forming pili (BFP) and genes encoding for shiga toxins. The aim of this study was to isolate, identify and determine virulence and antibiotic resistance profiles of aEPEC O177 strains from cattle feces. A total of 780 samples were collected from beef and dairy cattle and analyzed for the presence of *E. coli* O177. One thousand two hundred and seventy-two (1272) presumptive isolates were obtained and 915 were confirmed as *E. coli* species. Three hundred and seventy-six isolates were positively confirmed as *E. coli* O177 through amplification of *rmlB* and *wzy* gene sequences using multiplex PCR. None of these isolates harbored *bfpA* gene. A larger proportion (12.74%) of the isolates harbored *hlyA* gene while 11.20, 9.07, 7.25, 2.60, and 0.63% possessed *stx*_2_, *stx*_1_*, eaeA, stx*_2*a*_, and *stx*_2*d*_, respectively. Most of *E. coli* O177 isolates carried *stx*_2_/*hlyA* (9.74%). Furthermore, 7.40% of the isolates harbored *stx*_1_/*stx*_2_ while 7.09% possessed *stx*_1_/*stx*_2_/*hlyA* genes. Only one isolate harbored *stx*_1_/*stx*_2_/*hly*/*eaeA*/*stx*_2*a*_*/stx*_2*d*_ while 5.11% of the isolates harbored all the four major virulence genes *stx*_1_/*stx*_2_/*hlyA*/*eaeA*, simultaneously. Further analysis revealed that the isolates displayed varied antimicrobial resistance to erythromycin (63.84%), ampicillin (21.54%), tetracycline (13.37%), streptomycin (17.01%), kanamycin (2.42%), chloramphenicol (1.97%), and norfloxacin (1.40%). Moreover, 20.7% of the isolates exhibited different phenotypic multi-drug resistance patterns. All 73 isolates harbored at least one antimicrobial resistance gene. The *aadA, streA, streB, erm*, and *tetA* resistance genes were detected separately and/or concurrently. In conclusion, our findings indicate that environmental isolates of aEPEC O177 strains obtained from cattle in South Africa harbored virulence and antimicrobial resistance gene determinants similar to those reported in other shiga-toxin producing *E. coli* strains and suggest that these determinants may contribute to the virulence of the isolates.

## Introduction

Enteropathogenic *E. coli* (EPEC) is a group of diarrhoeagenic *E. coli* that is reported to cause high morbidity and mortality in humans, especially in immune-compromised subjects, elderly individuals and young children. EPEC are characterized by the presence of intimin (*eae*) genes coupled with the absence of the *stx* genes (Martins et al., 2016; Alonso et al., 2017). The *eae* gene is responsible for attaching and effacing (A/E) lesions on the intestinal epithelial cell of the host (Kaper et al., 2004; Martins et al., 2016; Malik et al., 2017). Based on the presence or absence of the EPEC adherence factor (EAF) plasmid, EPEC is subdivided into two groups that include typical Enteropathogenic *E. coli* (tEPEC) and atypical Enteropathogenic *E. coli* (aEPEC) (Trabulsi et al., 2002; Alonso et al., 2017). The tEPEC strains possess EAF plasmid, which encodes a bundle forming pili (*bfp*A) while aEPEC strains lack the *bfpA* gene (Canizalez-Roman et al., 2013; Malik et al., 2017). It is on these bases that the virulence potentials of aEPEC is poorly understood and highly questioned. Despite the fact that tEPEC have been most often associated disease complications in humans, it is only recently that aEPEC been reported to cause diseases in both animals and humans (Malik et al., 2017). This may account for the reason why previous studies that have been documented worldwide and in the study area have focused on EHEC, especially *E. coli* O157 and non-O157 strains that received great attention due to its high pathogenicity (Ateba and Bezuidenhout, 2008; Ateba and Mbewe, 2011; Iwu et al., 2016; Jajarmi et al., 2017; Toro et al., 2018). The findings of most of these studies were in agreement with previous reports indicating that EHEC strains possessed *stx, stx*_2_, *hlyA*, and *eaeA* gene determinants that enhance their potential to cause infections such as diarrhea, hemorrhagic colitis (HC) and hemolytic uremic syndrome (HUS) in humans (Farrokh et al., 2013). However, to the best of our knowledge there is currently no report on the occurrence of EPEC, and aEPEC in particular among South African food-producing animals. This therefore creates a knowledge gap on the virulence profiles of aEPEC strains in the area. Despite the fact that aEPEC strains were known to be less pathogenic when compared to EHEC counterparts, data from some recent studies have revealed the presence of virulence determinants in aEPEC strains thus making them to start receiving attention as pathogens of severe clinical significance in humans (Beutin et al., 2005; Bibbal et al., 2017) as well as in food-producing animals, especially sheep and wildlife (Otero et al., 2013; Álvarez-Suárez et al., 2016; Martins et al., 2016). In this study, we expand our previous investigations on aEPEC in the area by determining the occurrence of aEPEC O177 strains in cattle and also provide an overview of the putative virulence and antimicrobial resistance profiles of the isolates. This was motivated from the fact that cattle are the primary reservoir of both EHEC (*stx*^+^) and aEPEC (*stx*^−^) strains, thus providing opportunities for horizontal transfer of virulence and antibiotic resistant genes between species and/or strains living in the same ecological niches (Bibbal et al., 2017).

## Materials and Methods

### Samples Collection and Isolation of *E. coli* O177 Serogroup

A total of 780 cattle fecal samples were collected from eight commercial dairy and beef cattle farms in the North West Province, South Africa. Ethical clearance was obtained from the North West University Research Ethics Committee prior to the commencement of the study. Sampling was done between January 2017 and July 2017. The selection of the farms was based on the production system; either intensive, semi-intensive, and/or extensive farming system in the study area. The selection of all the three systems was based on the fact their management system is different. Furthermore, this was to avoid biasness in terms of selecting the farms. Ethical procedures such as restraining the animals using proper facilities and equipment were enforced during the collection of the samples. Fecal samples were collected directly from the rectum of individual animals using sterile arm-length gloves and in order to avoid duplication of sampling, the cattle were looked in their respective handling pens immediately after collection. Samples were placed in sterile sample collection bottles, labeled appropriately and immediately transported on ice to the Antimicrobial Resistance and Phage Biocontrol Laboratory (AREPHABREG), in the Department of Microbiology, North-West University for microbial analysis. Data on antibiotic type and treatment history were collected for the purpose of understanding antibiotic exposure histories of isolates from the study population.

Approximately, 2 g of fecal samples were dissolved in 10 mL of 10% (w/v) saline solution and homogenized. Aliquots of 5 μL were transferred into 10 mL buffered peptone water (Oxoid Ltd., Basingstok, Hampshire, UK). Ten-fold serial dilutions were prepared and aliquots of 100 μL from each dilution was spread-plated on Rainbow agarO157 plates (Kase et al., 2015) obtained from Biolog Inc., USA. The plates were incubated aerobically at 37°C for 24 h. Colonies with purple and pink colors were randomly picked and purified by streaking on sorbitol MacConkey agar (Merck, S.A) supplemented with 1 mg/L potassium tellurite (Merck, SA) and incubated aerobically at 37°C for 24 h. Pure isolates were preserved in 20 % (v/v) glycerol (Merck, SA) and stored at −80°C for future use.

### Genomic DNA Extraction From Presumptive Isolates

Overnight culture for each sample was prepared and genomic DNA was extracted from the cultures using the Zymo Research Genomic DNA^TM^-Tissue MiniPrep Kit (Biolab, South Africa) following the manufacturer's instructions. DNA concentration and purity was determined using the NanoDrop Lite 1,000 spectrophotometer (model: Thermo Fisher Scientific, USA) and the DNA was stored at −80°C until further analysis by PCR.

### Identification of *E. coli* O177 Serogroup Using Multiplex PCR Assay

A singleplex PCR assay targeting the *uidA E. coli* O177-specific gene sequence was performed using a previous protocol (Anbazhagan et al., 2011). *Escherichia coli* O177 serogroup-specific primer pairs were designed using the Primer3 software (this study), based on *rmlB* and *wzy* gene sequences that encode for dTDP-glucose 4, 6-dehydratase, and O-antigen polymerase, respectively (Ye et al., 2012). The specificity of the PCR primers was tested using National Center for Biotechnology Information/Primer-Basic Alignment Search Tool (NCBI/Primer-BLAST) (https://www.ncbi.nlm.nih.gov/tools/primer-blast/). The oligonucleotides were synthesized and supplied by Inqaba Biotechnical Industry Ltd., Pretoria, South Africa. The newly developed multiplex PCR protocol was empirically validated for its specificity, sensitivity reproducibility, and robustness. PCR assays was performed to amplify *rmlB* and *wzy* gene fragments and the primer sequences, targeted genes, amplicon sizes as well as the PCR conditions are listed in Table 1. The PCR reactions constituted 12.5 μL of 2X DreamTaq Green Master Mix, 0.5 μM of each primer and 1 μL of template DNA. The volume of the reaction mixture was adjusted to 25 μL with RNase-nuclease free PCR water. A non-DNA template (nuclease-free water) reaction tube served as a negative control while a DNA sample from *E. coli* O177 (isolated during the preliminary study) was used as positive control. All the PCR reagents used were Fermentas USA products supplied by Inqaba Biotec Industry Ltd., Sunnyside, Pretoria, South Africa. Amplifications were performed using DNA thermal cycler (model- Bio-Rad C1000 Touch^TM^ Thermal Cycler, Singapore). All the PCR products were held at 4°C until gel electrophoresis.

**Table 1:** Oligonucleotide primers used for amplification of the various targeted genes in *E. coli* O177 serogroup.

**Primers**	**Sequence**	**Target genes**	**Amplicon size (bp)**	**PCR conditions**	**PCR volume (25 μL)**	**References**
**I-IDENTIFICATION**						
UidA-F	CTGGTATCAGCGCGAAGTCT	*uidA*	600	95°C, 10 min(1x); 95°C, 45 s; 59°C, 30 s; 72°C, 90 s (35x); 72°C for 10 min (1x)	12.5 μL of 2X DreamTaq Green Master Mix, 11 μL of nuclease free water, 0.5 μL of each primer and 1 μL of template DNA	Anbazhagan et al., 2011
UidA-R	AGCGGGTAGATATCACACTC					
RmlB-F	CGCGGATTTTTGCTCTGCAT	*rmB*	645	95°C, 3 min (1x); 94°C, 30 s; 55°C, 30 s; 72°C, 60 s (30x); 72°C for 5 min (1x)	12.5 μL of 2X DreamTaq Green Master Mix, 11 μL of nuclease free water, 1 μL (0.5 μL of each) primer and 1 μL of template DNA	This study
RmlB-R	CAGTAATTGCGAGCCGCTTC					
Wzy-F	GGTCAGGAGCATGGAGCATT	*wzy*	457			
Wzy-R	AATCCATCCGGTGTATCGGC					
**II-VIRULENCE GENES**						
Bfp-F	AAT GGT GCT TGC GCT TGC TGC	*bfp*	324	95°C, 3 min (1x); 94°C, 60 s; 64°C, 60 s; 72°C, 2 min (35x); 72°C for 10 min (1x)	12.5 μL of 2X DreamTaq Green Master Mix, 11 μL of nuclease free water, 0.5 μL of each primer ad	Ghanbarpour et al., 2017
Bfp-R	GCC GCT TTATCC AAC CTG GTA					
EaeA-F	GACCCGGCACAAGCATAAGC	*eaeA*	384	95°C, 10 min (1x); 95°C, 60 s; 62°C, 2 min; 72°C, 90 s (35x), 72°C, 5 min (1x)		Paton and Paton, 1998
EaeA-R	CCACCTGCAGCAACAAGAGG				1 μL of template A	
HlyA-F	GCATCATCAAGCGTACGTTCC	*hlyA*	534	95°C, 10 min (1x); 95°C, 60 s; 64°C, 2 min; 72°C, 90 s (35x), 72°C, 5 min (1x)		
HlyA-R	AATGAGCCAAGCTGGTTAAGCT					
Stx1-F	ATAAATCGCCATTCGTTGACTAC	*stx_1_*	180	95°C, 10 min (1x); 95°C, 60 s; 62°C, 2 min; 72°C, 90 s (35x), 72°C, 5 min (1x)		
Stx1-R	AGAACGCCCACTGAGATCATC					
Stx2-F	GGCACTGTCTGAAACTGCTCC	*stx_2_*	255			
Stx2-R	TCGCCAGTTATCTGACATTCT					
Stx2a-F	AGATATCGACCCCTCTTGAA	*stx_2*a*_*	969	94°C, 5 min (1x); 94°C, 45 s; 60°C, 45 s; 72°C, 90 s (25x); 72°C, 7 min (1x)	12.5 μL of 2X DreamTaq Green Master Mix, 11 μL of nuclease free water, 0.5 μL of each primer and 1 μL of template DNA	He et al., 2012
Stx2a-R	GTCAACCTTCACTGTAAATG					
Stx2-G2-F	TATACGATGACACCGGAAGAAG	*stx_2*b*_*	300	94°C, 5 min (1x); 94°C, 45 s; 65°C, 30 s; 72°C, 60 s (25x); 72°C for 5 min (1x)		
Stx2-G2-R	CCTGCGATTCAGAAAAGCAGC					
Stx2/2c	TTTTATATACAACGGGTA	*stx_2*c*_*	163	94°C, 5 min (1x); 94°C, 45 s; 51°C, 30 s; 72°C, 60 s (30x); 72°C, 5 min (1x)		
Stx2-G1-R	GGCCACTTTTACTGTGAATGTA					
Stx2d-act	CTTTATATACAACGGGTG	*stx_2*d*_*	359	94°C, 5 min (1x); 94°C, 60 s; 50°C, 60 s; 72°C, 60 s (25x); 72°C, 5 min (1x)		
CKS2	CTGAATTGTGACACAGATTAC					
Stx2-G4-F	CAGGAAGTTATATTTCCGTAGG	*stx_2*e*_*	911	94°C, 5 min (1x); 94°C, 30 s; 55°C, 30 s; 72°C, 60 s (25x); 72°C, 5 min (1x)		
Stx2-G4-R	GTATTCTCTTCCTGACACCTTC					
Stx2-G3-F	TTTACTGTGGATTTCTCTTCGC	*stx_2*f*_*	875	94°C, 5 min (1x); 94°C, 30 s; 61°C, 30 s; 72°C, 60 s (25x); 72°C for 5 min (1x)		
Stx2-G3-R	TCAGTAAGATCCTGAGGCTTG					
209-F	GTTATATTTCTGTGGATATC	*stx_2*g*_*	573	94°C, 5 min (1x); 94°C, 45 s; 55°C, 60 s; 72°C, 60 s (25x); 72°C for 7 min (1x)		
781-R	GAATAACCGCTACAGTA					

### Detection of Virulence Genes in *E. coli* O177 Isolates

*E. coli* O177 isolates were subjected to a *bfp* gene PCR analysis in order to screen for characters of aEPEC strains. A highly virulent environmental *E. coli* O157:H7 isolate (*bfp*^+^) previously isolated from our research group (AREPHABREG) was used as positive control. The positive control *E. coli* O157:H7 possessed the *stx*_1_, *stx*_2_*, eaeA*, and *hlyA* genes. In addition, DNA extracted from an *E. coli* (ATCC 98222) that is a non-pathogenic strain was included in each PCR run as a negative control. Isolates that were positive for the *bfp* gene were further screened for the presence of an array of STEC virulence genes that included *eaeA, hlyA, stx*_1_, *stx*_2_, and *stx*_2_ variants (*stx*_2__a_, *stx*_2b_, *stx*_2c_, *stx*_2d_, *stx*_2e_, *stx*_2f_, *stx*_2*g*_) using previously described protocols (Paton and Paton, 1998; He et al., 2012). Primer sequences, target genes, amplicon sizes as well as PCR cycling conditions for the different genes are listed in Table 1. All the PCR reactions were prepared as 25 μL standard volumes comprising 12.5 μL of 2X DreamTaq Green Master Mix, 0.5 μM of each primer, 1 μL of template DNA and RNase-nuclease free PCR water. Amplifications were performed using DNA thermal cycler (model- Bio-Rad C1000 Touch^TM^ Thermal Cycler, Singapore). All the PCR products were held at 4°C until they were separated by electrophoresis.

### Antimicrobial Susceptibility Test

The antimicrobial susceptibility profile of the isolates was determined using the Kirby-Bauer disc diffusion technique (Bauer et al., 1966), making use of antibiotic discs obtained from Mast Diagnostics, UK. The antibiotics using in this study comprised Ampicillin (AMP), 10 μg; Chloramphenicol (C), 30 μg; Erythromycin (E), 15 μg; Kanamycin (K), 30 μg; Norfloxacin (NOR), 10 μg; Streptomycin (S), 10 μg and Tetracycline (TE), 30 μg. Some of these antimicrobial agents were selected due to the fact that they are widely used as prophylactics in both beef and dairy cattle farming in South Africa. Plates were incubated aerobically at 37°C for 24 h and antibiotic growth inhibition zone diameter data were compared with standard reference values in order to classify the isolates as sensitive, intermediate resistance or resistant to a particular antibiotic (Clinical Laboratory Standards Institute, 2016). *Escherichia coli* ATCC 25922 was used as a reference for quality control in antimicrobial susceptibility test.

### Detection of Genetic Determinants for Antibiotic Resistance Genes by PCR

All confirmed *E. coli* O177 isolates that were resistant to three or more antibiotics were designated multi-drug resistant isolates and were screened for the presence of the *tetA, tetW, aadA, strA, strB, ampC, cmlA, ermB*, and *kan* antibiotic resistance determinants. Primer sequences, target genes, amplicon sizes as well as PCR cycling conditions for the different genes are listed in Table 2. All the PCR reactions were prepared as 25 μL standard volumes comprising 12.5 μL of 2X DreamTaq Green Master Mix, 0.5 μM of each primer, 1 μL of template DNA and RNase-nuclease free PCR water. Amplifications were performed using DNA thermal cycler (model- Bio-Rad C1000 Touch^TM^ Thermal Cycler, Singapore). All the PCR products were kept at 4°C and later resolved by electrophoresis.

**Table 2:** Oligonucleotide primers used for amplification of the various targeted antibiotic resistance genes in *E. coli* O177 serogroup.

**Primers**	**Sequence**	**Target genes**	**Amplicon size (bp)**	**PCR conditions**	**PCR volume (25 μL)**	**References**
AadA-F	GTGGATGGCGGCCTGAAGCC	*aadA*	525	95°C, 3 min (1x); 94°C, 30 s; 55°C, 30 s; 72°C, 60 s (35x), 72°C, 5 min (1x)	12.5 μL of 2X DreamTaq Green Master Mix, 11 μL of nuclease free water, 0.5 μL of each primer and 1 μL of template DNA	Srinivasan et al., 2007
AadA-R	AATGCCCAGTCGGCAGCG					
StrA-F	CCTGGTGATAACGGCAATTC	*strA*	549			
StrA-R	CCAATCGCAGATAGAAGGC					
StrB-F	ATCGTCAAGGGATTGAAACC	*strB*	509			
StrB-R	GGATCGTAGAACATATTGGC					
CmlA-F	CCGCCACGGTGTTGTTGTTATC	*cmlA*	698			
CmlA-R	CACCTTGCCTGCCCATCATTAG					
AmpC-F	CATATGCTTAATCAGTGAGGCACCT	*ampC*	850	95°C, 3 min (1x); 95°C, 60 s; 59°C, 60 s; 72°C, 2 min (35x), 72°C, 10 min (1x)	12.5 μL of 2X DreamTaq Green Master Mix, 11 μL of nuclease free water, 0.5 μL of each primer and 1 μL of template DNA	Samra et al., 2009
AmpC-R	GAATTCAGTATTCAACATTTCCGTGTCG					
Kan-F	CATATGAGAAAAACTCATCGAGCATC	*kan*	810			
Kan-R	GAATTCAGCCATATTCAACGGGAA					
TetA-F	GCTACATCCTGCTTGCCTTC	*tet(A)*	210	95°C, 3 min (1x); 94°C, 30 s; 58°C, 30 s; 72°C, 60 s (35x); 72°C, 5 min (1x)	12.5 μL of 2X DreamTaq Green Master Mix, 11 μL of nuclease free water, 0.5 μL of each primer and 1 μL of template DNA	Bergeron et al., 2015
TetA-R	CATAGATCGCCGTGAAGAGG					
TetW-F	GAGAGCCTGCTATATGCC AGC	*tet(W)*	168			
TetW-R	GGGCGTATCCACAATGTTAAC					
ErmB-F	GATACCGTTTACGAA ATTGG	*ermB*	364	95°C, 60 s; 94°C, 15 s; 56°C, 30 s; 72°C, 60 s (40x) 72°C, 5 min	12.5 μL of 2X DreamTaq Green Master Mix, 11 μL of nuclease free water, 0.5 μL of each primer and 1 μL of template DNA	
ErmB-R	GAATCGAGACTTGAGTGTGC					

### Agarose Gel Electrophoresis

All PCR amplicons were resolved by electrophoresis on a 2% (w/v) agarose gel containing 0.001 μg/mL ethidium bromide. A 100 bp DNA molecular weight DNA marker (Fermentas, USA) was included in each gel and used to confirm the sizes of the amplicons. A ChemiDoc Imaging System (Bio-Rad ChemiDoc^TM^ MP Imaging System, UK) was used to capture the images using Gene Snap software, version 6.0022.

### Sequence Analysis

The amplified *rmlB* and *wzy* gene fragments were sequenced by Inqaba Biotec, Pretoria, South Africa, and sequences were subjected to a Blast Search Tool (https://blast.ncbi.nlm.nih.gov/Blast.cgi) in order to confirm the identities of the isolates (Altschul et al., 1997). Nucleotide sequence data were further deposited into GenBank using the BankIt NCBI web-based sequence submission tool (https://www.ncbi.nlm.nih.gov/genbank/) in order to obtain their accession numbers.

### Statistical Analysis

Measured parameters were tested for normality using the NORMAL option in the Proc Univariate statement prior to analysis of variance. Data on the proportions of samples positive for *E. coli* O177 serogroup were analyzed for effect of farming system using the general linear models (GLM) procedures of SAS (2010) according to the following statistical model:

$$\begin{array}{l} Y_{ij}=\mu +F_{i}+E_{ij} \end{array}$$

where, *Y*_*ij*_, response variable, μ, overall mean, F_*i*_, farming system (intensive, semi-intensive and extensive) effect and *E*_*ij*_, random error associated with observation *ij*, assumed to be normally and independently distributed. Data on number of isolates carrying virulent genes, number of antibiotic resistant isolates, number of isolates carrying antibiotic resistance genes, and number of multidrug resistant isolates were square root-transformed before statistical analysis using the GLM procedures (SAS, 2010). For all statistical tests, significance was declared at *p* < 0.05.

## Results

### Identification of *E. coli* O177 Serogroup Using Multiplex PCR Analysis

**Figure 1:**
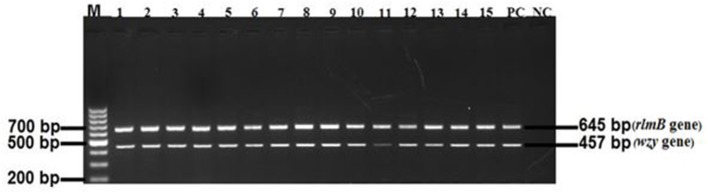
A 2% agarose gel image of the *rmlB* (645 bp) and *wzy* (457 bp) gene fragments amplified from *E. coli* O177 isolates. Lane M = 100 bp molecular marker; Lanes 1–15 = *rmlB* and *wzy* gene fragments, respectively, that were amplified from *E. coli* O177 isolates; Lane PC = *rmlB* and *wzy* gene fragments amplified from *E. coli* O177 serotype; Lane NC = negative control sample with DNA from *E. coli* O157:H7 environmental isolate.

A total of 780 cattle fecal samples were analyzed for the presence of *E. coli* O177 using Rainbow agarO157 and a total of 1,272 non-repetitive presumptive isolates were obtained. Amplification of the *uid*A *E. coli* genus-specific gene sequence revealed that a large proportion (915; 71.93%) of the isolates were successfully identified as *E. coli* isolates. All the 915 *E. coli* isolates were further screened for the presence of *E. coli* O177 serogroup specific *rmlB* and *wzy* gene fragments using multiplex PCR analysis. Out of 915 isolates screened, 376 (41.09%) were identified as *E. coli* O177 serogroup and Figure 1 indicates a 2% (w/v) agarose gel of representative *rmlB* and *wzy* gene fragments amplified in the study. No significant (*p* > 0.05) differences were observed on the occurrence of *E. coli* O177 serogroup across intensive, semi-intensive, and extensive animal production systems. The occurrence of *E. coli* O177 serogroup was 33.3 ± 14.43, 33.3 ± 14.43, and 50.0 ± 14.18% in intensive semi-intensive extensive farming systems, respectively.

### Detection of Virulence Genotypes in *E. coli* O177 Isolates

**Figure 2:**
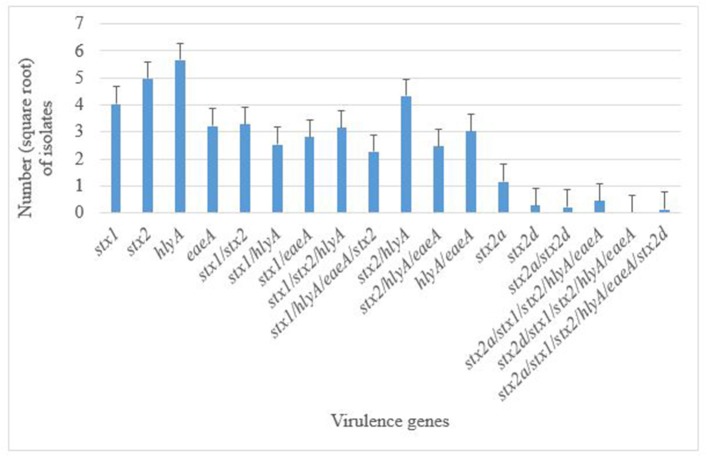
Distribution of virulence genes in *E. coli* O177 serogroup isolated from cattle feces. The bars indicate the standard error (*p* < 0.05).

A total of 376 atypical *E. coli* O177 isolates that were positively confirmed by PCR were screened for the presence of five virulence genes; *bfpA, eaeA, hlyA, stx*_1_, and *stx*_2_, using PCR analysis. None of the isolates possessed the *bfpA* gene fragment and thus were classified as aEPEC strains. On the contrary, all the other four virulence genes (*eaeA, hlyA, stx*_1_, and *stx*_2_) were successfully detected in this study. Generally, 350 isolates harbored these virulence genes. There were significant differences (*p* < 0.001) in terms of occurrence of various virulence genes in *E. coli* O177 isolates. The *hlyA* (12.74%) and *stx*_2_ (11.20%) were the most commonly detected genes (*p* < 0.001), followed by *stx*_1_ (9.07%), and *eaeA* (7.25%), Figure 2. Two *stx*_2_ subtypes namely; the *stx*_2a_ and *stx*_2*d*_, were detected among the isolates. A few of the isolates (2.60%) harbored *stx*_2*a*_, while 0.63% possessed *stx*_2*d*_gene. Some of the isolates carried a combination of the genes detected. The majority (9.74%) of the isolates carried a combination of *hlyA/stx*_2_ while 7.40, 7.09, 6.80, and 5.55% possessed *stx*_1_*/stx*_2_, *stx*_1_*/stx*_2_*/hlyA, hlyA/eaeA*, and *stx*_2_*/hlyA/eaeA*, respectively (*p* < 0.001). There was no difference (*p* > 0.05) between the occurrences of *stx*_1_/*eaeA* (6.35%) and *stx*_1_/*stx*_2_/*hlyA*/*eaeA* (5.11%) in *E. coli* O177 isolates. Only one isolate possessed *stx*_1_/*stx*_2_/*stx*_2*a*_/*stx*_2*d*_/*hlyA*/*eaeA*. Despite the fact that none of the isolates possessed the *stx*_2_ subtypes *stx*_2b_, *stx*_2c_, *stx*_2e_, *stx*_2f_, and *stx*_2g_, the virulence gene profiles of these aEPEC strains especially the isolate with the *stx*_2_ subtypes was a cause for concern.

### Antimicrobial Resistance Profiles

**Figure 3:**
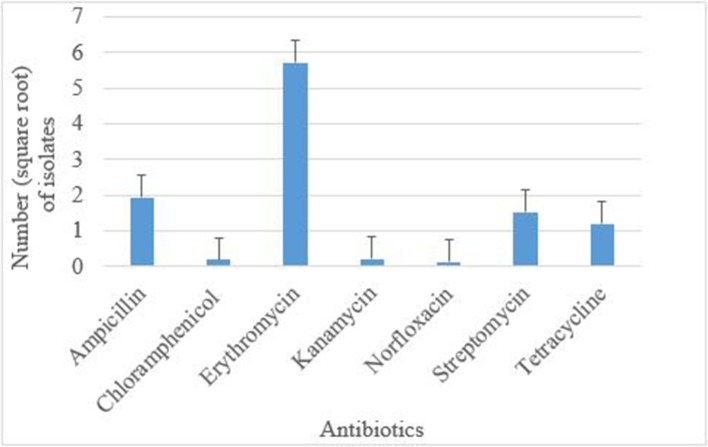
Antibiotic resistance pattern of *E. coli* O177 serogroup isolated from cattle feces. The bars indicate the standard error (*p* < 0.05).

**Figure 4:**
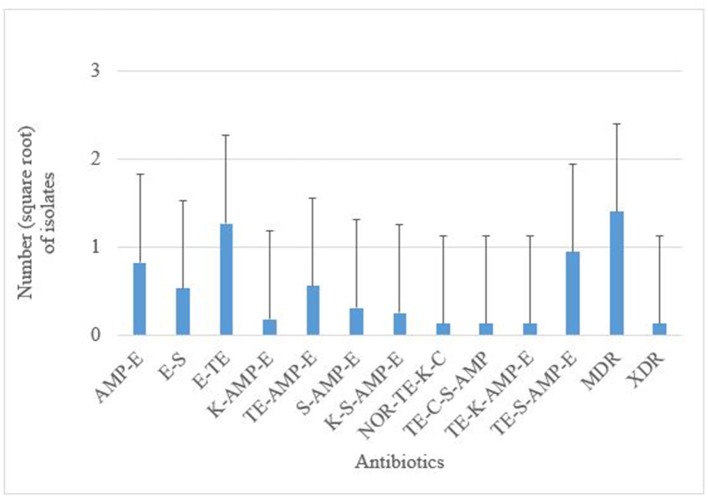
AMP, ampicillin; C, chloramphenicol; E, erythromycin; K, kanamycin; NOR, norfloxacin; TE, tetracycline; S, streptomycin; MDR, multidrug-resistant; XDR, extensively drug-resistant. Multiple resistance patterns of *E. coli* O177 serogroup obtained from cattle feces. The bars indicate the standard error (*p* < 0.05).

Antimicrobial susceptibility tests of the 376 isolates revealed varied antimicrobial resistance profiles against all the seven antibiotics tested. Most of the *E. coli* O177 isolates were resistant to erythromycin (63.84%) compared to other antibiotics (*p* < 0.001), Figure 3. No difference (*p* > 0.05) was observed in *E. coli* O177 resistance to ampicillin (21.54%), tetracycline (13.37%), streptomycin (17.01%), kanamycin (2.42%), chloramphenicol (1.97%), and norfloxacin (1.40%). The isolates showed resistance to at least two antibiotics tested. Most of the isolates (20.74%) exhibited high level of resistance to three or more antibiotics, Figure 4 (*p* < 0.001).

### Proportion of Isolates With Antimicrobial Resistance Genes

A total of 73 *E. coli* O177 isolates harbored either one or more different antimicrobial resistance genes investigated, however, only five of the ten genes were detected in the isolates. Resistance genes associated with streptomycin and erythromycin were the most frequently detected. Genes *aadA* (22.86%), *strA* (19.85%), *strB* (19.38%) *ermB* (19.68%), and *tetA* (18.23%) were detected. However, there was no significant differences in the occurrence of these genes in *E. coli* O177 isolates. No isolates possessed the *tetW* gene. Some isolates harbored a combination of three or more antibiotic resistance genes. Most (24%) of the isolates carried a combination of the *aadA, strA*, and *strB* genes while a smaller proportion 11% isolates possessed all the five antimicrobial resistance genes (*aadA*/*strA*/*strB*/*ermB*/*tetA*) (*p* < 0.05).

### Sequence Identifier and Accession Numbers

Sequence data analysis of the *rmlB* and *wzy* gene fragments amplified from chromosomal DNA of isolates revealed a high percentage similarity (97%) sequence homology to *E. coli* serogroup O177. The nucleotide sequences for Seq1, Seq2, and Seq3 were assigned GenBank Accession numbers; MH389799, MH389800, and MH389801, respectively.

## Discussion

In this study, the occurrence of aEPEC, *E. coli* O177 serogroup in cattle was investigated. Although the virulence profiles of aEPEC strains is poorly understood, recent reports suggest increased incidence of human infections caused by aEPEC strains worldwide (Ingle et al., 2016; Martins et al., 2016). Cattle are primary reservoir of *E. coli* species and contaminated meat as well as their associated food products have been implicated in foodborne infections in humans (Ateba and Mbewe, 2011). Studies have reported the occurrence of aEPEC strain in food-producing animals, foods of animal origin and farm environments (Horcajo et al., 2012; Otero et al., 2013; Ghanbarpour et al., 2017). *Escherichia coli* was successfully isolated from cattle feces using Rainbow agar O157. The identities of the isolates were confirmed as *E. coli* through amplification of *uidA* gene fragments. The r*mlB* and *wzy* O-antigen gene clusters specific for *E. coli* O177 were successfully amplified through Multiplex PCR analysis. The PCR assay was highly specific and reproducible for the detection of *E. coli* O177 serogroup from cattle feces. The occurrence of *E. coli* O177 in cattle was higher (41.09 %) in this study when compared to a previous report which detected this serogroup in ovine (Martins et al., 2016). Despite this, there were no significant (*p* > 0.05) differences on the occurrence of *E. coli* O177 serogroup across all the three farming systems.

Given the fact that aEPEC strains are known to lack medically important virulence factors, their ability to cause diseases particularly in humans is not yet well-understood (Ingle et al., 2016). Previous reports indicated that aEPEC strains do not harbor *stx* and *bfpA* genes. However, recent reports have revealed that aEPEC strains especially those from ruminants, harbor virulence gene profiles similar to those of the EHEC strains (Horcajo et al., 2012). In addition, some studies have reported that aEPEC strains may represent LEE-positive Shiga toxin-producing *E. coli* (STEC) that have lost the toxin-encoding prophage, or tEPEC that have lost the genes for BFP indicating the potential to have evolved from other pathogenic *E. coli* strains (Tennant et al., 2009). A motivation was the fact that previous studies conducted in the study area revealed a large proportion of virulent EHEC strains (Ateba and Bezuidenhout, 2008; Ateba and Mbewe, 2011, 2013, 2014). Despite the fact that we were unable at this stage to determine if aEPEC isolated from humans in South African cattle fall into either of these categories, an assessment of the *bfpA* and other primary STEC virulence genes (*eaeA, hlyA, stx*_1_, *stx*_2_, and *stx*_2_ variants) was performed. Data of the shiga toxin subtypes among non-O157 STEC serogroups in cattle may provide an indication of the potential health risks these aEPEC isolates may cause humans. The *bfpA* gene was not detected in all the *E. coli* isolates analyzed and thus confirmed that the isolates belonged to aEPEC group, emerging diarrheagenic pathogens in both developing and developed countries (Ingle et al., 2016; González et al., 2017).

All the major virulence genes (*stx*_1_, *stx*_2_, *hlyA*, and *eaeA*) were detected in this study. Similar findings were reported by Momtaz et al. (2013a) and Momtaz et al. (2013b). Generally, the majority of *E. coli* O177 isolates possessed virulence genes. The *hlyA* was the most frequently detected virulence gene in this study. These results are similar to those reported in other studies (Ateba and Bezuidenhout, 2008; Ateba and Mbewe, 2011; Momtaz et al., 2013c), which described high prevalence of *hlyA* gene in *E. coli* O157 isolated from cattle, beef, vegetable and water intended for human consumption. The high prevalence of *hlyA* gene may be attributed to the fact that *hlyA* is a plasmid encoded gene and as a result, it may be transferred between strains of the same species (Ateba and Mbewe, 2011). The *hlyA* gene encodes α-hemolysin, a toxin that lyses mammalian erythrocytes (Toro et al., 2018).

Another observation was that the *E. coli* O177 isolates possessed *stx* genes. These genes are considered as primary virulence genes in shiga toxin-producing *E. coli* and their presence in aEPEC strains, especially *E. coli* O177 serogroup raises a serious public health concern. The *stx*_2_ was the most prevalent gene detected in STEC/EHEC (Ateba and Mbewe, 2011; Rantsiou et al., 2012). Despite the fact that low frequency of *stx*_2_ gene was observed in this study, the occurrence of this gene was significantly higher than *stx*_1_ (*p* < 0.001). This observation was similar to the findings of the previous studies (Beutin et al., 2005; Jajarmi et al., 2017; Toro et al., 2018). Interestingly, *stx*_2_ positive isolates also harbored *stx*_2*a*_ and *stx*_2*d*_ gene subtypes. However, the other *stx*_2_ subtypes were not detected. Despite this, the presence of *stx*_2_, *stx*_2*a*_, and *stx*_2*d*_ genes in *E. coli* O177 serogroup is a serious concern. Strains harboring these genes have been implicated in hemolytic colitis and hemorrhagic uremic syndrome infections in humans (Farrokh et al., 2013). It is also reported that a strain harboring *stx*_2_ gene is more virulent than a strain carrying either *stx*_1_ or both *stx*_1_ and *stx*_2_ (Farrokh et al., 2013; Toro et al., 2018). Moreover, *stx*_2*a*_ and *stx*_2*d*_ subtypes are associated with severe HC and HUS infections in human (Farrokh et al., 2013; Cha et al., 2018). Therefore, the presence of *stx*_2_gene and its subtypes in *E. coli* O177 may increase the spectrum of infections in humans and thus creating a public health concern.

The *stx*_1_ and *eaeA* genes were also detected but at low levels compared to *hlyA* and *stx*_2_ gene fragments. This was in contrast with the previous studies, which reported high occurrence of *stx*_1_ and *eaeA* genes in *E. coli* isolated from poultry meat and humans (Momtaz et al., 2013b; Hemmatinezhad et al., 2015). Despite low detection of these genes, especially *E. coli* O177 serogroup, these findings cannot be overemphasized. The strain carrying *stx*_1_ may cause diarrhea in immunocompromised individuals (Farrokh et al., 2013). In addition, the *eaeA* gene encodes for the outer membrane which mediates adherence between the STEC and/or EPEC and intestinal epithelial cells (Kaper et al., 2004; Farrokh et al., 2013). Although *eaeA* was detected in this study, this gene was not detected in some isolates. This was surprising because all the isolates were negative for *bfpA* gene and thus classified as aEPEC strains. These results differ significantly with other studies where the *eaeA* gene has been detected in aEPEC strains (Trabulsi et al., 2002; Beutin et al., 2005; Otero et al., 2013; Martins et al., 2016; Ghanbarpour et al., 2017; González et al., 2017). The possible explanation for this observation could be that the isolates may have lost the gene during sub-culturing process (Karch et al., 1992). Given that the strains carrying *eaeA* gene are considered potentially pathogenic, absence of this gene in some of the isolates in this study should not be underestimated. The *eaeA*-negative isolates may use other genes such as *saa, aidA, agn43, ehaA*, or *iha* to adhere to the epithelial cell of the host (Toro et al., 2018). However, the presence of these genes was not investigated in this study.

The co-expression of *stx*_1_, *stx*_2_, *hlyA*, and *eaeA* genes may increase the pathogenicity of *E. coli* strains (Jajarmi et al., 2017). It was observed that *E. coli* O177 isolates possessed combinations of virulence genes. There were twelve different combinations of virulence genes detected in this study. The most frequently detected combinations were *stx*_2_/*hlyA* (9.74 %), *stx*_1_/*stx*_2_ (7.40%), *stx*_1_/*stx*_2_/*hlyA* (7.09%), and *hlyA*/*eaeA* (6.80%). Only 5.11% of the isolates harbored all the four genes (*eaeA*/*hlyA*/*stx*_1_/*stx*_2_). It was also remarkable that one isolate possessed all the virulence (*stx*_1_/*stx*_2_/*hlyA*/*eaeA*) genes and two *stx*_2_ (*stx*_2*a*_ and *stx*_2*d*_ genes) subtypes. Simultaneous detection of these virulence genes in *E. coli* O177 serogroup in cattle pose a public health concern. Furthermore, these findings are similar to those reported in *E. coli* O26 in a previous study (Jajarmi et al., 2017; Ranjbar et al., 2017). The combinations of virulence genes in this study are higher than those reported by the previous studies (Ateba and Mbewe, 2011; Jajarmi et al., 2017; Toro et al., 2018). However, there was low occurrence of combination of *stx*_2_ subtypes as compared to a previous study (Jajarmi et al., 2017).

Antimicrobial agents primarily play a vital role in the lives of both humans and animals worldwide (Hudson et al., 2017). South Africa, is one of the countries where the usage of antimicrobial agents in animal production is very high (Hudson et al., 2017). In addition, the overuse of antimicrobial agents in food-producing animals, often without either professional consultation or supervision, has resulted in the emergence of antimicrobial resistant bacteria and antibiotic resistant genes in human and animal pathogens (Qiao et al., 2017). Moreover, previous investigations in the study area revealed the presence of antimicrobial resistant gene determinants in *E. coli* O157 isolated from cattle, beef, pig, pork, vegetables, and water intended for human consumption (Ateba and Bezuidenhout, 2008). Despite this, farmers continue to use antimicrobial agents to maximize production and this presents severe public health challenges.

In this study, *E. coli* O177 isolates revealed phenotypic resistance against all the seven classes of antimicrobial agents tested. In contrary to the previous study which reported high prevalence of resistance against ampicillin (100%), gentamicine (100%), and tetracycline (96.87%) Ranjbar et al. (2018), this study revealed resistance against erythromycin (63.84%), ampicillin (21.54%), tetracycline (13.37%), streptomycin (17.01%), and kanamycine (2.42%). These disparities could be attributed to geographical location and the sample type. Furthermore, 20.74% of the isolates were resistant to at least three and antimicrobial agents tested. These results were similarly to those reported in the previous study (Ranjbar et al., 2017). Antimicrobial resistance genes encoding for three antibiotics (erythromycin, streptomycin, and tetracycline) were detected. It has been reported that antibiotic resistance is common among *E. coli* isolates obtained from animals and food of animal origin due to frequent use of antibiotics in animals (Ryu et al., 2012). Generally, all isolates harbored antibiotic resistance genes. It is also worth mentioning that the same isolates harbored STEC virulence genes and previous studies have shown that isolates with similar genetic determinants may pose severe complications in humans (Ateba and Bezuidenhout, 2008; Hemeg, 2018). The occurrence of antibiotic resistance genes detected in this study was higher as compared to the previous study (Ryu et al., 2012). This could be due to the relatively higher usage of antimicrobial agents in the intensive livestock farming system where the isolates were obtained.

Antibiotic resistance genes associated with some of the antibiotics tested in this study were detected. The aadA*, strA*, and *strB* were the most frequently detected genes. However, there were significant differences among all the genes. Most of the isolates possessed *aadA* (22.86%), *strA* (19.85%), and *strB* (19.38%) genes that code for resistance to streptomycin and similar observations have been reported in other studies (Srinivasan et al., 2007; Shahrani et al., 2014; Bibbal et al., 2017). Despite the fact that *tet* genes are commonly found in *E. coli* from cattle due to the high usage of tetracycline in dairy and feedlot cattle, the occurrence of *tet* resistance genes in *E. coli* O177 isolates was low. Only the *tetA* gene was detected individually or in combination with other genes. However, in a previous report (Olowe et al., 2013), the proportion of *tet*A (43.8%) was higher than *tetB* (32.0%) and a combination of both *tetA* and *tetB* (4.4%) among clinical *E. coli* isolates. In addition, Ranjbar et al. (2018) reported *tetA* (76.56%), *tetB* (20.31%), *cat1* (18.75%) and *cmlA* (1.50%) in *E. coli* O157 and non-O157 serogroups from milk. Another study reported high detection of *tetA* and *tetB* in *E. coli* isolated from urinary infection patients, ruminants and donkey raw milk (Momtaz et al., 2012, 2013c). Similar to a previous report (Olowe et al., 2013), none of the isolates in this study harbored the *tetW, amp, cml*A, and *kan* genes.

In conclusion, to the best of our knowledge this is the first study to report the occurrence of *E. coli* O177 strain in cattle, especially in South Africa. In this study, the search for virulence determinants in aEPEC clearly revealed that a majority of strains harbored DNA sequences that encode known virulence-associated determinants of other pathogenic *E. coli* such as O157 and non-O157 strains. In addition, *E. coli* O177 strains from this study displayed high levels of resistance to aminoglycoside antibiotic group thus have the potential to pose a public health concern to humans. Indeed, the fact that all aEPEC strains in this study expressed a variety of tEPEC virulence determinants supports the case for continued efforts to conduct a large scale study designed to determine the phylogenetic homology among aEPEC isolates from different sources. Furthermore, whole genome sequencing of *E. coli* O177 is required in order to understand the complete pathogenic determinants of this serogroup.

## Data Availability Statement

The datasets generated for this study can be found in the NCBI.

## Author Contributions

VM and CA contributed to project conception and contributed reagents, materials, and analysis tools. PM, VM, and CA designed the experiments. PM performed the experiments. All authors contributed to manuscript revision and read and approved the submitted version.

### Conflict of Interest

The authors declare that the research was conducted in the absence of any commercial or financial relationships that could be construed as a potential conflict of interest.
